# Evaluation of Heterogeneous Nuclear Ribonucleoprotein D Expression as a Diagnostic Marker for Oral Squamous Cell Carcinoma

**DOI:** 10.3390/diagnostics12061332

**Published:** 2022-05-27

**Authors:** Vikas Kumar, Moien Rasheed Lone, Anurag Kumar, Vinnyfred Vincent, Himani Thakkar, Deepika Mishra, Shyam S. Chauhan

**Affiliations:** 1 Department of Biochemistry, All India Institute of Medical Sciences, New Delhi 110029, India; vksalhan@aiims.edu (V.K.); lonemoien49@gmail.com (M.R.L.); anuraginsewagram@gmail.com (A.K.); drvincent2520@gmail.com (V.V.); himanithakkar88@gmail.com (H.T.); 2 Division of Oral Pathology, Centre for Dental Education and Research, All India Institute of Medical Sciences, New Delhi 110029, India; deepikamishra@aiims.edu

**Keywords:** hnRNPD, p40, diagnostic marker, oral cancer

## Abstract

The heterogeneous nuclear ribonucleoprotein D (hnRNPD) serves as a prognostic marker for oral squamous cell carcinoma (OSCC). We evaluated the diagnostic potential of hnRNPD to differentiate between OSCC and normal mucosa. Immunohistochemistry for hnRNPD and a routinely used diagnostic marker deltaNp63 (p40) was performed in 32 normal mucosae and 46 OSCC specimens. Subsequently, receiver-operating characteristic analysis was performed to evaluate the diagnostic potential of hnRNPD in comparison to that of p40. Immunostaining for p40 and hnRNPD was observed in 39 (84.78%) and 38 (82.60%) cases, respectively, in OSCC specimens. The poorly differentiated squamous cell carcinoma displayed 100% (eight cases) immunoreactivity for hnRNPD as compared to 87.5% (seven cases) for p40. Nuclear staining of p40 and hnRNPD was observed in all OSCC specimens. p40 staining was restricted to basal cells, whereas both basal and para-basal cells displayed hnRNPD staining in OSCC specimens. Areas under the curve for p40 and hnRNPD were 0.86 and 0.87, respectively. p40 and hnRNPD showed equal sensitivities (80.95%). However, hnRNPD displayed marginally higher (88.23%) specificity for tumor cells as compared to that of p40 (85.29%). Conclusion: In addition to being a well-established prognostic marker, hnRNPD can serve as a diagnostic marker for OSCC.

## 1. Introduction

Oral squamous cell carcinoma (OSCC) is the most common type of malignancy that arises in the oral cavity. The majority of OSCC occurrences are of squamous cell origin and account for more than 90% of malignancies of the oral cavity [1]. The overall survival of patients with this disease is poor, despite advancements in treatment strategies [2]. This is because most cases are only diagnosed at advanced stages. The most common screening modality for oral cancer is based on inspection for visual alterations in oral mucosa, followed by a biopsy for histological grading. These histological alterations are based on changes in architectural and cytological features of epithelial cells [3,4,5,6]. The diagnostic markers routinely used in pathological laboratories include cytokeratins, p16, tumor protein 63 (p63), and p40 [7,8,9]. The ΔNp63 isoform is transcribed from p63, which is also known as p40. This isoform is specific for squamous epithelial cells [10]. Overexpression of p40 is frequently reported in cancers of squamous cells in origin that include lung, prostate, urothelium, and oral cancer [11,12].

Regulation of gene expression by alteration of mRNA stability and translation in response to various intracellular and extracellular stimuli is a key feature of eukaryotes. Approximately 16% of protein-coding human genes contain adenylate uridylate-rich elements (ARE) in the 3’UTR of their mRNA [13,14]. Heterogeneous nuclear ribonucleoprotein D (hnRNPD), also known as adenylate uridylate-rich element RNA-binding factor 1(AUF1), is the first identified and most studied RNA binding protein [15]. The human hnRNPD gene, containing 9-exons and 8-introns, is located on chromosome 4q21. The alternative splicing of exons 2 and 7 results in the generation of four different isoforms: p37, p40, p42, and p45. These are named according to the molecular weights of proteins encoded by these isoforms [16,17]. hnRNPD plays a critical role in inflammation, alternative splicing, tumorigenesis, and apoptosis by regulation of mRNA stability [18,19,20,21]. Over-expression of hnRNPD has been reported in thyroid, lung, colon, esophageal, breast, and oral cancers [22,23]. Additionally, one previous report demonstrated a strong association between nuclear expression of hnRNPD and poor prognosis in oral cancer patients and suggested its diagnostic utility in this malignancy [22]. In the present study, we compare the diagnostic potential of hnRNPD with p40, a diagnostic marker used to differentiate OSCC from normal mucosa, and our results convincingly demonstrate that hnRNPD can serve as a diagnostic marker for OSCC, in addition to p40.

## 2. Material and Methods

### 2.1. Tissue Specimens

After obtaining approval from the Institute Ethics Committee for Post Graduate Research, All India Institute of Medical Sciences, New Delhi, India (NO. IECPG-162/19.04.2018), previously prepared paraffin-embedded tissue specimens were used in the present study. Before sample collection, all participants provided written informed consent, which is a mandatory requirement from the institute’s ethics committee. All experiments were performed following guidelines issued by the committee. A total of 46 oral cancer tissue specimens were utilized in the present study. Normal (non-dysplastic) mucosa from 32 healthy individuals who underwent tooth extraction served as controls. All relevant information regarding patient samples, including confirmed TNM staging, histopathological differentiation status, and socio-demographical parameters, were obtained from the department of pathology, AIIMS (Table 1 and Table 2).

### 2.2. Immunohistochemical Staining

Paraffin-embedded tissue sections were deparaffinized before antigens were retrieved. Then, endogenous peroxidase activity was quenched with hydrogen peroxide (0.3% *v*/*v*) and non-specific binding was blocked with 1% bovine serum albumin (BSA). The slides were then incubated with either rabbit monoclonal anti-hnRNPD (clone: D6O4F, Cell Signaling Technology, Danvers, MA, USA) antibody or mouse monoclonal anti-p40 (clone: YN0123m, Elabscience, Houston, TX, USA) for 16 h at 4 °C. The primary antibody was detected using the Dako Envision kit (Dako CYTOMATION, Glostrup, Denmark), with diaminobenzidine as the chromogen, and counterstained with hematoxylin. The sections were evaluated by light microscopy and scored using a semi-quantitative scoring system for both staining intensity (nuclear/cytoplasmic) and percentage positivity. We used the mean value of percentage positivity in five randomly selected areas of tissue sections. Expressions of both proteins were scored independently by two pathologists blinded to the identity of sections and their scores. The tissue sections were scored based on the percentage of immunostained cells as: 0–10% = 0; >10–30% = 1; >30–50% = 2; >50–70% = 3; and >70–100% = 4. Sections were also scored semi-quantitatively on the basis of staining intensity as negative = 0; mild = 1; moderate = 2; intense = 3. Finally, a total score was obtained by adding the score of percentage positivity and intensity, giving a score range from 0 to 7. A total score ≥2 was taken as positive and a total score of less than 2 was considered negative for analysis of hnRNPD and p40 expression.

### 2.3. Statistical Analysis

Statistical analysis was performed using R software (R Core Team 2021). (R: A language and environment for statistical computing. R Foundation for Statistical Computing, Vienna, Austria. URL https://www.R-project.org/ (accessed on 19 July 2021).) The receiver operator curve (ROC) for both hnRNPD and p40 was plotted using the “ROCit” package in R. Sensitivity, and specificity was calculated for various threshold values between −1 and infinity. A threshold of 0.5 provided optimum sensitivity and specificity for both the proteins. Sensitivity, specificity, positive predictive value (PPV), and negative predictive value (NPV) were calculated concerning this threshold. The McNemar test was used to compare the performance of hnRNPD and p40 as diagnostic markers. In the McNemar test, *p*-value < 0.05 indicates a significant difference in performance of the two diagnostic markers.

## 3. Results

### 3.1. Clinicopathological Features

The clinicopathological features of the specimens used in the present study are provided in Table 1 and Table 2. A total of 32 normal oral mucosa specimens were recruited, 26 of whom were males (81.25%) and 6 of whom were females (18.75%). Of the 46 oral cancer patients recruited in the present study, 38 patients were males (83.60%) and 8 were females (17.39%). The mean age of these patients at the time of diagnosis was 45 years (ranging from 22 to 75 years of age). These cases were histologically graded as well-differentiated (17; 36.95%), moderately differentiated (21; 45.65%), and poorly differentiated (8; 17.39% cases).

### 3.2. Immunohistochemical Analysis

**Figure 1:**
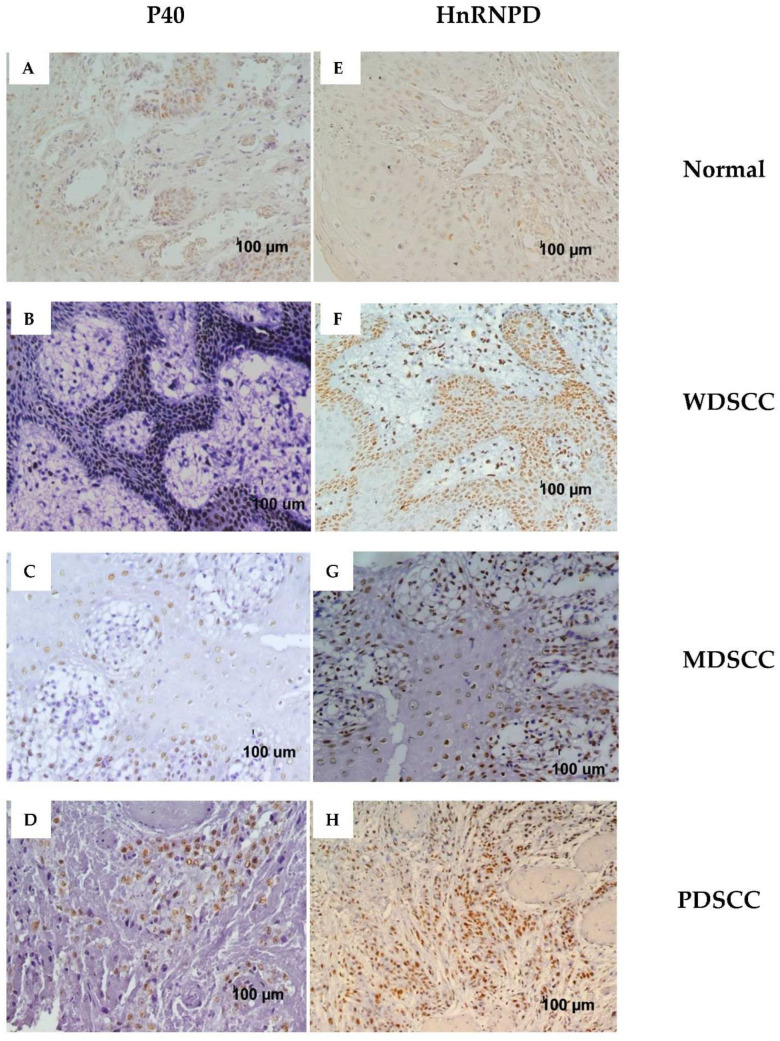
Immunohistochemistry of P40 (**A**–**D**) and hnRNPD (**E**–**H**). Normal tissue (**A**,**E**), WDSCC (**B**,**F**), MDSCC (**C**,**G**), and PDSCC (**D**,**H**) show a staining pattern of p40 and hnRNPD. Magnification for each photomicrograph is 200×.

p40 expression analysis in normal mucosa was found positive in 3 out of 32 specimens (9.37%) with mild nuclear immunostaining as represented in Figure 1A. However, immune-histochemical analysis of oral squamous cell carcinoma tissue specimens revealed that 39 (84.78%) out of 46 stained positives for p40 (Table 3). A total of 31 specimens out of 46 (67.39%) displayed moderate to intense nuclear staining for p40 (intensity score ≥ 2), out of which 11/31 (35.48%) patients showed immunostaining in more than 50% of tumor cells (Table 4). However, in seven (15.21%) cases, immunostaining for p40 was in the range of 0–10%, and hence it was considered to be negative. According to the histological grades of squamous cell carcinoma based on tumor differentiation, the positive staining for p40 was observed in 15/17 (88.23%) cases of well-differentiated squamous cell carcinoma (WDSCC; Figure 1B), 17/21 cases (80.95%) of moderately differentiated squamous cell carcinoma (MDSCC; Figure 1C) and 7/8 cases (87.50%) of PDSCC (Figure 1D) (Table 3). As is evident from Figure 1, p40 immunostaining was confined specifically to the nucleus of basal cells (Figure 1B–D).

Consistent with the immunoreactivity of p40, hnRNPD also showed a similar staining pattern for the normal oral mucosa, 2 out of 32 cases (6.25%) found to be positive with mild nuclear staining (Figure 1E). However, in the case of oral squamous cell carcinoma specimens, a total of 38 cases out of 46 (82.60%) displayed positive nuclear staining for hnRNPD in basal and parabasal cells (Figure 1F–H). The 2+ intensity score that represents moderate to intense staining was similar for both hnRNPD and p40. However, in the case of hnRNPD, there was an overall increase in the tumor cell’s immunoreactivity in 20/31 (64.51%) cases as compared to p40 (11/31; 35.48%). A total of eight (17.39%) cases were negative for hnRNPD staining. The tumor differentiation-based staining was positive in 15 (88.23%; Figure 1F) WDSCC, 15 (71.42%; Figure 1G) MDSCC, and 8 (100%; Figure 1H) PDSCC cases.

**Figure 2:**
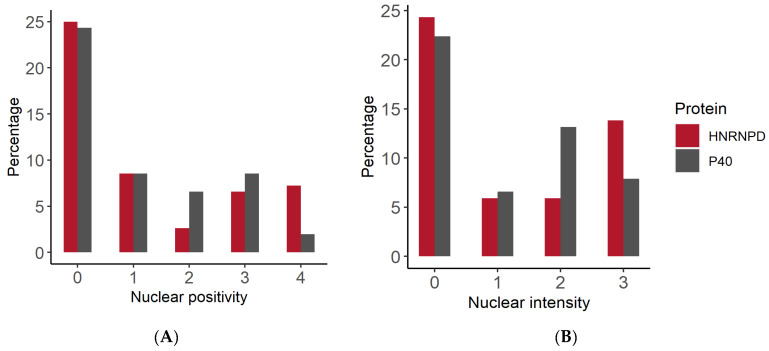
Immunohistochemistry score analysis of p40 and hnRNPD in OSCC. (**A**) Nuclear immunoreactivity score. (**B**) Nuclear intensity score.

**Figure 3:**
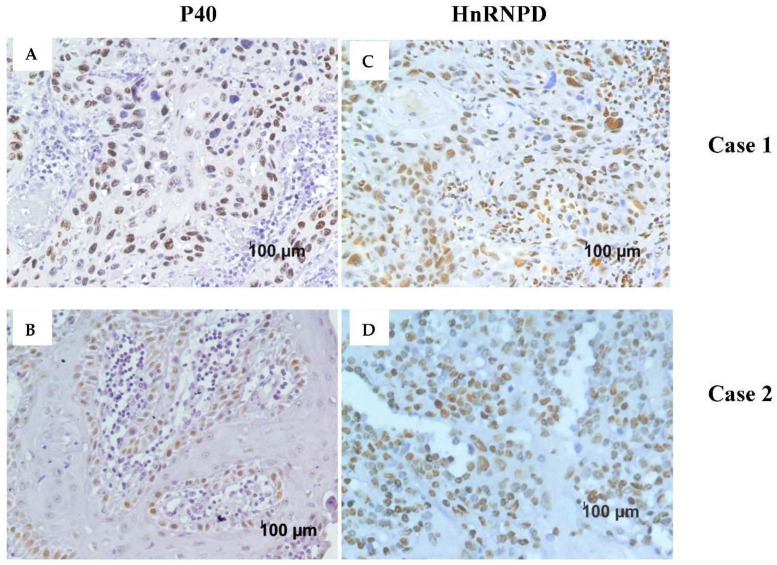
p40 (**A**,**B**) and hnRNPD (**C**,**D**) immunohistochemistry. Few patient samples show high tumor reactivity for hnRNPD compared to p40, as seen in Case1 (**A**,**B**) and Case2 (**C**,**D**).

Thus, both p40 and hnRNPD proteins showed nuclear immunostaining in 38 out of 46 squamous cell carcinoma specimens. Seven of the remaining eight specimens were stained negative for both hnRNPD and p40. However, one specimen displayed mild staining for p40 but turned out to be negative hnRNPD. These results conclusively demonstrate that 82.60% of oral squamous cell carcinoma tissues stained positive for both p40 and hnRNPD. Representative immunostaining for p40 and hnRNPD in different tumor grades is given in Figure 1. As evident from these results, hnRNPD showed intense staining in 23 (50%) cases with more than 70% tumor cells reactivity in 11/23 (47.82%) cases. In contrast, only 12/46 (26.08%) specimens showed intense p40 staining and >70% tumor cells reactivity only in 2/12 (16.66%) specimens (Figure 2). These results suggest hnRNPD (Figure 3C,D) to be a more specific marker of squamous cell carcinoma as compared to p40 (Figure 3A,B).

**Figure 4:**
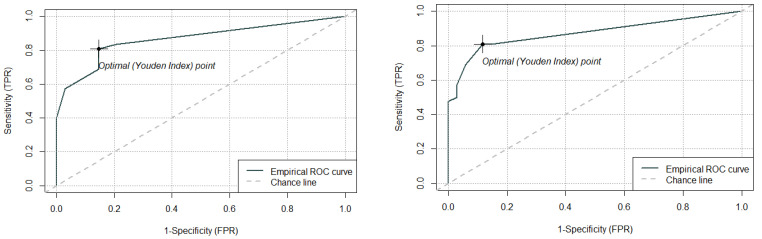
Receiver-operating characteristic (ROC)–area under curve (AUC) analysis to evaluate the diagnostic potential of hnRNPD. (**A**) P40 AUC (0.86). (**B**) hnRNPD AUC (0.87).

Receiver-Operating Characteristic (ROC) analysis was performed to evaluate the diagnostic potential of hnRNPD in comparison with p40. The area under the curve (AUC) for p40 and hnRNPD was found to be 0.86 and 0.87, respectively (Figure 4). The p40 and hnRNPD showed equal sensitivity (80.95%) for squamous cell carcinoma, whereas hnRNPD emerged as marginally more specific than p40 with 88.23% specificity and 89.47% positive predictive value in comparison to p40 with 85.29% specificity and 87.17% positive predictive value (Table 5). The McNemar test comparing the performance of hnRNPD and p40, which had a *p*-value of 0.54, indicated no significant difference between the performance of the two markers in differentiating between normal and malignant tissue. Thus, our results suggest that hnRNPD, in addition to p40, could serve as a new independent diagnostic marker for OSCC.

## 4. Discussion

Apart from incorporating the latest innovation in therapeutic interventions, upgrading and introducing new diagnostic and prognostic markers holds the key to better overall management of any disease, including cancer. In current practice, several diagnostic markers for oral cancers such as cytokeratin, p63, p40, and p16 [7,8,9,24,25] have been established. For nominating any novel target as a probable addition to the list of high-quality diagnostic markers, a wide range of analyses is warranted to ascertain its analytical utility and diagnostic accuracy. A study involving a proteomics-based approach identified numerous protein markers, namely prothymosin alpha (PTMA), S100A7, 14-3-3ζ, 14-3-3δ, hnRNPD, and hnRNPK, used for distinguishing between normal mucosa, dysplasia, and oral cancer [26,27]. Over-expression of nuclear hnRNPD in oral cancer compared to that present in normal oral tissues is associated with poor prognosis [22]. These findings compel us to believe that hnRNPD can serve as a potential candidate for the diagnosis of oral cancer.

TAp63 and ΔNp63/p40 are encoded by two different mRNA isoforms transcribed from the same gene (TP63) and located on chromosome 3q28 by alternate promoters [28]. TAp63 isoform contains an N-terminal transactivation domain that is absent in ΔNp63 [29]. This transactivation domain activates numerous targets of the p53 gene and thus performs a tumor-suppressor role similar to p53 [28]. The lack of transactivation domain in the p40 isoform makes it an oncoprotein [29]. It is only detected in squamous cells, and its expression increases gradually from normal to oral dysplasia being highest in head and neck squamous cell carcinoma [9]. p40 mediates up-regulation of keratin 6A, 14 and cancer stem cell marker CD44, which promotes abnormal differentiation of basal epithelial cells that lead to oral squamous cell carcinoma [10,30]. It also promotes tumor cell survival [31]. p40 has been used as a specific marker to differentiate squamous cell carcinoma from other malignancies such as sarcoma, etc. [11,32]. Given these reports, we carried out a systematic study to evaluate and compare the diagnostic potential of hnRNPD with p40, a specific marker for squamous epithelial cells.

For the first time, the results of the present study demonstrate hnRNPD to be more specific and a better marker than p40, which is used to distinguish cases of OSCC from normal counterparts. The finding of this study unearths almost comparable sensitivity of p40 and hnRNPD in diagnosing oral squamous cell carcinoma. Both p40 and hnRNPD are located in the nucleus of squamous cell carcinoma cells of the oral cavity, which is in agreement with previous reports [12,22]. The normal tissue specimens showed mild to negligible nuclear immunostaining for both p40 and hnRNPD only in a few cases. The overall positivity for OSCC specimens is comparable for both p40 and hnRNPD staining. The poorly differentiated squamous cell carcinoma tissue specimens showed 100% positivity for hnRNPD staining in comparison to p40 (87.50% positivity). The present study’s results agree with one of the previous reports where hnRNPD displayed 81.1 to 88.23% specificity and 65.5 to 80.95% sensitivity in diagnosing OSCC [22]. hnRNPD showed overall improvement in immunohistochemical staining in terms of tumor-specific reactivity and intensity. p40 staining was limited to basal cells, which is similar to previous reports [9,12,33]. Consistent with the previous finding, hnRNPD was detected in the nuclei of basal cells [22]. Interestingly, in addition to basal cells, we observed moderate to intense staining of hnRNPD in parabasal cells of squamous epithelium. Both p40 and hnRNPD displayed equal sensitivity, but hnRNPD exhibited an improved overall specificity as compared to p40. Thus, based on the findings of the present study, we put forward hnRNPD as a new diagnostic marker for OSCC, in addition to a prognostic marker. Therefore, we propose the candidature of hnRNPD as an additional diagnostic marker, marginally superior to p40 in OSCC.

## Figures and Tables

**Table 1:** Characteristics of normal and oral squamous cell carcinoma specimens (OSCC) used in evaluating the diagnostic potential of hnRNPD.

	Normal Mucosa (*n* = 32)	Oral Squamous Cell Carcinoma (OSCC) (*n* = 46)
**Sex**		
Male	26 (81.25)	38 (83.60)
Female	6 (18.75)	8 (17.39)
**Age**	46.31 ± 13.10	45 ± 13.18
**Histological grade**		
Well-differentiated	-	17
Moderately differentiated	-	21
Poorly differentiated	-	8

**Table 2:** Clinico-pathological characteristics of tissue specimens and total IHC score for p40 and hnRNPD.

Patients No.	Age/Gender	Tumor Site	Pathological Stage	Histological Stage	P40 Total Score *	HnRNPD Total Score *
**1**	75/M	Tongue	T3N0M0	PDSCC	4	2
**2**	55/M	Buccal mucosa	T4N1M0	MDSCC	0	0
**3**	40/F	Hard palate	T2N2M0	WDSCC	3	3
**4**	52/F	Tongue	T2N1M0	MDSCC	7	7
**5**	45/M	Tongue	T2N2M0	MDSCC	4	7
**6**	15/M	Buccal mucosa	T4N0M0	WDSCC	0	0
**7**	75/M	Buccal mucosa	T4N1M0	WDSCC	3	6
**8**	49/M	Tongue	T2N1M0	WDSCC	6	5
**9**	52/F	Buccal mucosa	T4N1M0	WDSCC	6	7
**10**	60/M	Retromolar trigone	T3N1M0	WDSCC	0	0
**11**	33/M	Buccal mucosa	T4N1M0	MDSCC	3	0
**12**	35/M	Retromolar trigone	T4N1M0	MDSCC	0	0
**13**	71/M	Tongue	T2N2M0	WDSCC	6	3
**14**	55/M	Tongue	T1N1M0	MDSCC	5	1
**15**	29/M	Buccal mucosa	T2N1M0	MDSCC	6	7
**16**	38/M	Buccal mucosa	T4N2M0	PDSCC	4	7
**17**	35/M	Retromolar trigone	T4N0M0	MDSCC	0	1
**18**	57/M	Buccal mucosa	T4N1M0	MDSCC	4	6
**19**	38/M	Buccal mucosa	T2N0M0	MDSCC	5	7
**20**	35/M	Buccal mucosa	T2N1M0	MDSCC	4	2
**21**	55/F	Alveolar	T4N1M0	MDSCC	0	0
**22**	45/M	Alveolar	T4N1M0	WDSCC	2	3
**23**	39/M	Buccal mucosa	T4N1M0	WDSCC	3	7
**24**	55M	Soft palate	T2N2M0	WDSCC	2	4
**25**	60/F	Buccal mucosa	T3N1M0	WDSCC	6	6
**26**	35/M	Retromolar trigone	T4N1M0	MDSCC	5	6
**27**	35/M	Retromolar trigone	T4N0M0	MDSCC	3	3
**28**	55/M	Alveolar	T4N2M0	MDSCC	6	2
**29**	48/M	Gingivobuccal sulci	T2N0M0	WDSCC	3	4
**30**	31/M	Tongue	T3N2M0	WDSCC	5	7
**31**	52/M	Tongue	T2N0M0	MDSCC	5	6
**32**	36/M	Buccal mucosa	T3N2M0	MDSCC	4	6
**33**	33/M	Tongue	T4N2M0	WDSCC	2	3
**34**	44/F	Tongue	T2N1M0	WDSCC	3	2
**35**	32/M	Alveolar	T4N2M0	MDSCC	6	6
**36**	35/M	Tongue	T1N0M0	PDSCC	1	4
**37**	31/M	Tongue	T4N0M0	WDSCC	6	7
**38**	55/M	Retromolar trigone	T4N1M0	MDSCC	3	5
**39**	48/M	Tongue	T4N1M0	MDSCC	4	6
**40**	53/M	Buccal mucosa	T3N1M0	WDSCC	6	6
**41**	60/F	Buccal mucosa	T3N1M0	MDSCC	7	6
**42**	29/F	Buccal mucosa	T3N1M0	PDSCC	2	4
**43**	62/M	Alveolar	T4N1M0	PDSCC	3	4
**44**	31/M	Buccal mucosa	T4N1M0	PDSCC	5	7
**45**	67/M	Buccal mucosa	T2N1M0	PDSCC	5	7
**46**	38/M	Buccal mucosa	T4N2M0	PDSCC	2	3

* Total score ≥ 2 was considered positive.

**Table 3:** p40 and hnRNPD expression in different histological grades of oral squamous cell carcinoma and normal mucosa tissue specimens.

	Normal Mucosa	Oral Squamous Cell Carcinoma (OSCC)	Histological Grade		
			WDSCC	MDSCC	PDSCC
**p40**					
Positive	3 (9.37)	39 (84.78)	15 (88.23)	17 (80.95)	7 (87.50)
Negative	29 (90.62)	7 (15.21)	2 (11.76)	4 (19.04)	1 (12.50)
**hnRNPD**					
Positive	2 (6.25)	38 (82.60)	15 (88.23)	15 (71.42)	8 (100)
Negative	30 (93.75)	8 (17.39)	2 (11.76)	6 (28.57)	0 (0)

**Table 4:** Immunoreactivity for p40 and hnRNPD in oral squamous cell carcinoma samples.

	Immunostained Cells					Intensity Score				
	No.	0	1	2	3	0–10%	10–30%	30–50%	50–70%	>70%
**p40**	46	7 (15.21)	8 (17.39)	19 (41.30)	12 (26.08)	8 (17.39)	12 (26.08)	9 (19.56)	14 (30.43)	3 (6.52)
**hnRNPD**	46	8 (17.39)	7 (15.21)	8 (17.39)	23 (50)	8 (17.39)	12 (26.08)	4 (8.69)	10 (21.73)	12 (26.08)

**Table 5:** Comparison of sensitivity and specificity of p40 and hnRNPD in oral squamous cell carcinoma.

	Sensitivity (%)	Specificity (%)	PPV (%)	NPV (%)
**p40**	80.95	85.29	87.17	78.37
**hnRNPD**	80.95	88.23	89.47	78.94

## Data Availability

Materials and raw data that support the findings of the present study can be made available from the corresponding author upon reasonable request.

## Abbreviations

hnRNPD: heterogeneous ribonucleoprotein D; p63: tumor protein 63; p40: tumor protein 40; ΔNp63: delta N terminal isoform of tumor protein 63; TAp63: transactivation domain of p63; OSCC: oral squamous cell carcinoma; ROC: receiver-operating characteristic; AUC: area under curve.
